# Alterations in the p53-SOCS2 axis contribute to tumor growth in colon cancer

**DOI:** 10.1038/s12276-017-0001-1

**Published:** 2018-04-06

**Authors:** Jong-Hwan Kim, Mi-Jin Lee, Goung-Ran Yu, Sang-Wook Kim, Kyu-Yun Jang, Hee-Chul Yu, Baik-Hwan Cho, Dae-Ghon Kim

**Affiliations:** 1 Division of Gastroenterology and Hepatology, Department of Internal Medicine, Research Institute of Clinical Medicine, Chonbuk National University Hospital and Medical School, Jeonju, Jeonbuk 54907 Republic of Korea; 2 Department of Pathology, Research Institute of Clinical Medicine, Chonbuk National University Hospital and Medical School, Jeonju, Jeonbuk 54907 Republic of Korea; 3 Department of General Surgery, Research Institute of Clinical Medicine, Chonbuk National University Hospital and Medical School, Jeonju, Jeonbuk 54907 Republic of Korea

## Abstract

Altered expression of suppressor of cytokine signaling (SOCS) is found in various tumors. However, regulation of SOCS2 by upstream molecules has yet to be clearly elucidated, particularly in tumor cells. SCOCS2 expression was examined in tumor cells transfected with an inducible p53 expression system. The impact of SOCS2 on cell proliferation was measured with in vitro assays. Inhibition of tumorigenicity by SOCS2 knockdown was assessed via a mouse model. Expression profiles were compared and genes differentially expressed were identified using four types of p53-null cells (Saos, HLK3, PC3, and H1299) and the same cells stably expressing p53. Twelve kinds of target genes were simultaneously upregulated or downregulated by p53 in three or more sets of p53-null cells. SOCS2 expression was reciprocally inhibited by inducible p53 expression in p53-null cells, even colon cancer cells. SOCS2 promoter activity was inhibited by wild type but not mutant p53. SOCS2 knockdown inhibited tumor growth in vitro and in an animal xenograph model. SOCS2 overexpression was detected in a murine model of azoxymethane/dextran sulfate sodium-induced colitis-associated colon cancer compared to mock-treated controls. SOCS2 expression was heterogeneously upregulated in some human colon cancers. Thus, SOCS2 was upregulated by p53 dysfunction and seemed to be associated with the tumorigenic potential of colon cancer.

## Colon cancer: A delicate balance to regulate growth

Insights into a signaling protein’s role in cell growth could inform new therapeutic strategies for treating colon cancer. SOCS-2 acts as an ‘off switch’ for cell signaling pathways. It has been identified as possibly protective against many cancers, although some cancers are associated with elevated SOCS-2 levels. Researchers led by Daeghon Kim at Chonbuk National University Hospital in South Korea have now shown that the effects of SOCS-2 are apparently dependent on how much of it is present. Moderate levels of SOCS-2 can suppress growth in colon cancer cells, but Kim’s team showed that excessive SOCS-2 has the opposite effect, promoting proliferation. The researchers also identified a gene commonly mutated in cancer cells that can drive overproduction of SOCS-2. Drugs that inhibit SOCS-2 or block its production may therefore offer useful treatments for colorectal cancer.

## Introduction

Cytokine signaling (SOCS) molecules are suppressed by cytokines and function as negative regulators of cytokine-triggered cell signaling. They act by attenuating cytokine action through inhibition of Janus kinase (JAK) signal transducer and activator of transcription (STAT)^1^. Eight proteins, SOCS1–7 and cytokine-inducible src homology-2 (SH2)-containing proteins, have been identified according to the structural domains of a C-terminal SOCS box, a central SH2 domain, and an N-terminal variable domain^2^. SOCS proteins bind to the molecular apparatus to prevent further signal transduction and for targeting of degradation via the SOCS box, by recruiting ubiquitin ligases. A small kinase inhibitory region is located in the N-terminal domain of SOCS1 and SOCS3^3^. Altered expression of SOCS1 and SOCS3 has been identified in human carcinomas and is derived from dysregulation of signals from cytokine receptors, toll-like receptors, and hormone receptors^4^. SOCS1 and SOCS3 are potent inhibitors of activation of the JAK–STAT pathway and play critical roles in various malignant processes^5–8^. SOCS2 expression is downregulated in lung cancer, hepatocellular carcinoma, and prostate cancer^9–11^. However, SOCS2 is highly upregulated and has tumor-promoting functions in the advanced stages of chronic myeloid leukemia and in high-grade anal intraepithelial lesions^12,13^. High SOCS2 expression is found in androgen-stimulated prostate cancer cells and is associated with pro-proliferation^14^. Also, upregulation of SOCS2 is recognized as a potential marker for prostate cancer prognosis^15^. Mice lacking SOCS2 exhibit accelerated postnatal growth, significant increases in bone and body length, and increases in internal organ size^16^, suggesting SOCS2 attenuates growth hormone (GH) signaling. However, transgenic mice overexpressing SOCS2 are not growth deficient and are significantly larger than wild-type mice^17^. Therefore, SOCS2 is recognized as having a dual role in GH signaling, depending on its concentration; specifically, SOCS2 is a positive regulator of growth at high levels. The molecular mechanism(s) of the growth-promoting effect of SOCS2 is proposed to be due to phosphorylated tyrosine 595 on the GH receptor at the SOCS2 interaction site^17^. However, the reason why SOCS2 is abundantly expressed in some tumors remains unclear.

In this study, we propose that SOCS2 expression is also regulated by oncogenic alteration of the gene. Disruption of the p53 signaling pathway, such as loss of p53 function, was involved in SOCS2 induction in tumor cells and subsequently promoted proliferative activity of the cells.

## Materials and methods

### Cell culture, transfection, and reagents

Human tumor cell lines SAOS, H1299, PC3, HT29, DLD-1, SW480, COLO205, and A293 cells were from the American Tissue Culture Collection (Rockville, MD). HLK3 cells were established and maintained in our laboratory^18^. Cells were cultured in DMEM medium (Sigma, St. Louis, MO) supplemented with 10% fetal bovine serum (Invitrogen, Carlsbad, CA), 1% penicillin and streptomycin solution (Sigma), 3 mM taurine, and 25 mM HEPES (Invitrogen) in air containing 5% CO_2_ in an incubator, as previously described^18^. HCT116 with p53 wild type and HCT116 that are p53 null were gifts from Dr. Bert Vogelstein (Johns Hopkins University, Baltimore, MD). p53-null cells were transfected with a fusion gene of wild-type p53 linked to a modified ligand-binding domain of the murine estrogen receptor (p53ER^TM^) and selected with 2 μg/ml puromycin for 2 weeks, as previously described^19^. 4-Hydroxytamoxifen was from Sigma.

### Illumina BeadChip array hybridization and data analysis

Total RNA from Saos, HLK3, PC3, and H1299 cells stably expressing p53 or mock was isolated using TRIzol reagent (Invitrogen) according to the manufacturer's instructions. Biotin-labeled cRNA samples for hybridization were prepared according to Illumina's recommended sample-labeling procedure: 500 ng of total RNA was used for cDNA synthesis, followed by an in vitro transcription amplification/labeling step to synthesize biotin-labeled cRNA using Illumina TotalPrep RNA Amplification kits (Ambion Inc., Austin, TX). Labeled, amplified material (1500 ng per array) was hybridized to Illumina Human-6 BeadChips v2 containing 48,701 probes for 24,498 genes, according to the manufacturer's instructions (Illumina, San Diego, CA). Array signals were developed by Amersham fluorolink streptavidin-Cy3 (GE Healthcare Bio-Sciences, Little Chalfont, UK) following the BeadChip manual. Arrays were scanned with an Illumina BeadArray Reader confocal scanner (BeadStation 500GXDW; Illumina) according to the manufacturer's instructions. Array data processing and analysis used Illumina BeadStudio software. The BeadStudio Gene Expression Module analyzes gene expression data from scanned microarray images generated by the Illumina BeadArray Reader. Genes with expression that was at least twofold different than the median gene expression level across all samples in at least 10% of samples were selected for cluster analysis. Average linkage hierarchical cluster analysis was carried out using Pearson correlation as the similarity metric, using the GeneCluster/TreeView program (http://rana.lbl.gov/EisenSoftware.htm). We used *t* test *P* = 0.01. To ascertain biological relevance, a fold-change cutoff value of 1.5 from the mean was chosen.

### Cell lysis, immunoblotting, and antibodies

Extracted protein (30 μg) from cell lysates was resolved by SDS-PAGE and transferred to a nitrocellulose membrane as previously described. Nineteen membranes were incubated overnight at 4 °C in primary antibody, washed twice with PBS/0.1% Tween, and incubated for 1 h in secondary antibody. Blots were washed twice with PBS/0.1% Tween and developed using commercial chemiluminescence detection kits (Amersham ECL, Buckinghamshire, UK). Polyclonal SOCS2 antibody was from Cell Signaling Technology (#2779, Danvers, MA). GFP (sc-8334), p53 (DO-1, sc-126), and p21 (C-19, sc-397) antibodies were from Santa Cruz Biotechnology (Dallas, TX). Alpha tubulin (T6199) was from Sigma-Aldrich Co. (St. Louis, MO).

### Immunohistochemistry and immunofluorescence

For immunohistochemistry (IHC), paraffin blocks were sliced into 4-µm sections and deparaffinized. SOCS2 protein in tumor tissue sections was detected with labeled streptavidin-biotin detection kits (DAKO, Glostrup, Denmark) after microwave antigen retrieval. Sections were incubated with anti-SOCS2 (Santa Cruz Biotechnology) diluted 1:200. Alternatively, IHC was performed on tissue array slides containing colon cancer tissue samples and normal colon tissue samples. Immunohistochemical staining of SOCS2 was evaluated based on staining intensity score and staining area score for each specimen. The intensity of cytoplasmic and membranous staining was graded as follows: no immunostaining (0), weak (1), moderate (2), or strong (3). The proportion of positive cells was scored as follows: 0 (none), 1 (1%), 2 (2–10%), 3 (11–33%), 4 (34–66%), and 5 (67–100%). The sum index was obtained by totaling the scores of intensity and proportion of staining. If the final score was equal to or greater than 4, immunoreactivity was considered positive. For negative controls, sections were treated in the same way, except that they were incubated with Tris-buffered saline instead of primary antibodies. Histological examinations were performed by an experienced pathologist who was blinded to clinical information. For immunofluorescence, cells were grown on glass coverslips, fixed with 4% paraformaldehyde, permeabilized in PBS containing 0.2% Triton X-100, and blocked with 1% BSA. Transient transfection of a GFP-tagged SOCS2 expression vector or empty vector into HCT116 (p53^+/+^) cells and SW480 cells were performed using Lipofectamine (Invitrogen) according to the manufacturer’s protocol. Cells were incubated with rabbit polyclonal antibody against SOCS2 overnight at 4 °C, washed, and incubated with tetramethylrhodamine isothiocyanate isomer R-conjugated anti-rabbit immunoglobulin. After a final wash, cells were stained for 15 min with 1 μg/ml Hoechst 33258 to visualize nuclei and mounted in 50% glycerol in PBS at 4 °C. Cells were examined by laser scanning microscopy (LCM 510, Carl Zeiss, Jena, Germany).

### Luciferase assays

HEK293T cells were transfected with a SOCS2-luc reporter construct using Lipofectin (Gibco**-**BRL, Grand Island, NY). SOCS2-luc reporter construct included PCR amplification of the promoter region of the SOCS2 gene (−1500 to +135 bp) in a PGL3B basic reporter. Luciferase activity was measured using the Dual-Luciferase Reporter Assay System (Promega, Madison, WI) according to the manufacturer’s instructions, and firefly luciferase readings were normalized to *Renilla* luciferase readings. Reporter plasmids and wild-type and mutant p53 expression plasmids were cotransfected into HEK293 cells.

### Chromatin immunoprecipitation

For crosslinking, formaldehyde was added directly to the culture medium of HCT116 (p53^+/+^) or vector control cells at a final concentration of 1%. Part of the supernatant was retained as total chromatin input and processed with eluted immunoprecipitates beginning at the crosslink reversal step. Rabbit polyclonal anti-p53 or mouse monoclonal anti-IgG_1_ (negative control) was added to precleared chromatin, which was rotated overnight at 4 °C. Immunocomplexes were as eluted by resuspending protein A-Sepharose (Amersham Biosciences) in elution buffer followed by a 45-min incubation at room temperature. DNA was extracted with phenol/chloroform and precipitated with ethanol. PCR was performed using Taq DNA polymerase (Promega) according to the manufacturer’s protocol with the following primers:

P4: 5′-AATACAAAGACCCTGAAGCAGGGGCAA-3′ and 5′-GCGCGGTGGCTCACGCCTGTAATCCCA-3′; P3: 5′-ACCGCGCCCGGCCAGGATTCTTTTAAT-3′ and 5′-AAGCAATTCTCCTGCCTCAGCCTCCCG-3′; P2: 5′-GTAATCCCAGCTTCTCGGGAGGCTGAG-3′ and 5′-GTTGAATAATTTTGCACAAGGCACTTA-3′;

 P1: 5′-AAATTATTCAACTACTTTGTAGAGGAT-3′ and 5′-GTTGAGGCCGCGGCTATGGGAAGTTGG-3′.

PCR products were analyzed on 2% agarose gels stained with ethidium bromide.

### Mouse tumorigenicity assays

Four-week-old female athymic nude mice (BALB/cByJ-*Hfh11*^*nu*^, Orient Co., South Korea) were used in all experiments. Animals were maintained in a specific pathogen-free environment. The animal room was kept at 20−22 °C under a 12-h light–dark cycle. HCT116 (p53^−/−^) cells were transduced with SOCS2 target lentivirus or nontarget lentivirus vector. Cells (5 × 10^6^ in 80 μl PBS) mixed with 20 μl Matrigel were injected subcutaneously into both shoulders of nude mice. Growth curves were plotted based on mean tumor volume within each experimental group at the indicated time points. Tumor measures were length and width. Tumor volume was calculated according to the equation: V (mm^3^) = width^2^ (mm^2^) × length (mm)/2. Tumor growth was observed for at least 3 weeks. In vivo tumorigenic experiments were performed in seven mice per treatment group.

### Knockdown experiments

A lentivirus vector encoding shRNA targeting SOCS2 and shRNA nontarget control were used to transduce HCT116 (p53^−/−^) and HCT116 (p53^+/+^) cells according to the manufacturer’s instructions (Sigma): 1.2 × 10^5^ cells were seeded on 6-well plates overnight, then transduced with lentiviral particles at 10 MOI in the presence of 8 μg/ml hexadimethrine bromide (Sigma).

### Colon cancer animal models

We used a murine model of azoxymethane (AOM)/dextran sulfate sodium (DSS)-induced colitis-associated colon cancer (CAC). Six-week-old male C57BL/6 mice (Orient Bio Inc., Seongnam, Korea) were housed under specific pathogen-free conditions with free access to laboratory chow (Cargill Agri Purina, Inc., Seongnam, Korea) and water. Ten mice were injected intraperitoneally with 7.4 mg/kg body weight AOM dissolved in physiological saline. Five days later, 3% DSS was administered in drinking water for 5 days, followed by 16 days of regular water. This cycle was repeated three times. Ten mice were injected intraperitoneally with saline and administered saline in drinking water as controls. Following killing, colons were removed and opened longitudinally. The number of macroscopic tumors was counted and measured using calipers. Subsequently, distal colons were fixed in 10% neutral-buffered formalin for 24 h and transferred to 70% ethanol for paraffin embedding and histological analysis.

### Tissue acquisition

Paired samples of tumors and corresponding nontumor tissues were obtained from resected colon specimens from patients with colon cancer. Written informed consent was obtained from all patients. Tissues derived from surgical resection were rinsed in sterile phosphate-buffered saline (PBS) and were immediately snap frozen and stored in liquid nitrogen. All protocols conformed to the ethical guidelines of the Institutional Review Board of Chonbuk National University Hospital.

### Statistical analysis

All cell-based experimental results are expressed as the mean ± SE of at least three independent experiments performed in duplicate. Statistical evaluations of numeric variables in each group were conducted using two-tailed *t* tests and analysis of variance. The Mann–Whitney *U* test and *χ*^2^ test were used for evaluation of immunohistochemical SOCS2 expression in tumor tissues. All statistical analyses were performed using SPSS software, version 18.0 (IBM SW, Cambridge, MA). Statistical significance was defined as a *P* value less than 0.05.

## Results

### Downregulation of SOCS2 by p53

**Fig. 1:**
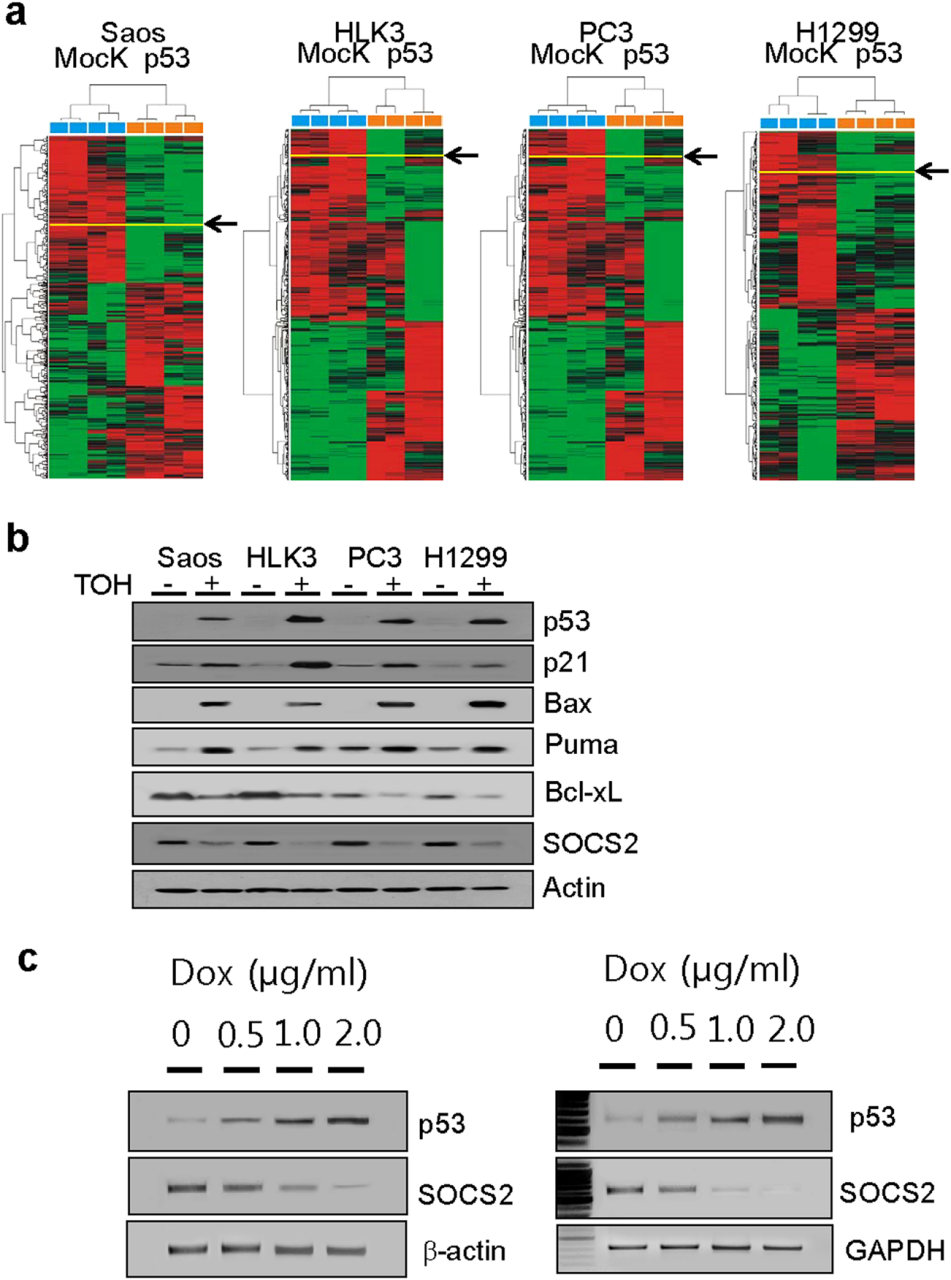
SOCS2 expression in p53-inducible cells. **a** Unsupervised hierarchical clustering separated samples into two groups: mock and p53. SOCS2 had a twofold or greater expression difference from mean at *P* < 0.05 based on t tests for hierarchical clustering. Data are presented in matrix format. Columns are individual cell lines and rows are genes. Red, high expression; green, low expression; black, no significant expression change between mean and sample. **b** Levels of SOCS2 and various p53 target proteins were analyzed by immunoblot of four p53-inducible lines (Saos, HLK3, PC3, and H1299). p53-null cells were transfected with inducible p53 plasmids (p53ER^TM^) and immunoblot analysis after 5 µM 4-OH tamoxifen, 24 h. **c** Protein (left panels) and mRNA expression levels (right panels) of SOCS2 were inversely regulated by endogenous p53 expression in HCT116 p53(+/+) cells. Dox doxorubicin

Unsupervised hierarchical clustering analysis of paired cells of LacZ-expressing p53-null and p53-expressing cells was based on similarities in expression patterns for all genes (Fig. 1a). Samples were clustered into two main groups: a mock group (p53-null cells) and a p53 group (p53-expressing cells). From the two major sample clusters, genes with a *P* value <0.05 and with a mean difference of expression >1.5 between groups were selected (Table 1). Specific immunoreactivity against SOCS2 and its cytoplasmic localization was confirmed by western blot and immunofluorescence assays (Supplementary Figure 1). *SOCS2* expression was reduced in p53-expressing cells compared with mock cells in four cell lines. Downregulation of *SOCS2* is associated with p53 expression. We determined SOCS2 protein levels in four kinds of paired cells using western blots. SOCS2 expression was reduced in cells in which p53 expression was inducible by treatment with tamoxifen, compared with vehicle-treated cells. p53-expressing cells also expressed p53 target proteins including p21, Bax, Puma, and Bcl-xL (Fig. 1b). These results suggested that p53 expression sufficiently downregulates expression of SOCS2. To examine whether endogenous p53 expression also affects SOCS2 expression, we treated HCT116 p53(+/+) cells with doxorubicin, a well-known p53 inducer, and found that doxorubicin efficiently induced p53 protein and mRNA expression in a dose-dependent manner. Subsequently, SOCS2 expression was inversely regulated in cells in a dose-dependent manner (Fig. 1c).

**Table 1:** Selected genes differentially expressed between p53-expressing and p53-null cells

Clone IC	Annotation	Saos	HLK3	PC3	H1229
ILMN_2131861	SOCS2	Down	Down	Down	Down
ILMN_2188862	GDF15	UP	UP	UP	UP
ILMN_1659047	HIST2H2AA3	UP	UP	UP	ns
ILMN_1678757	BCYRN1	ns	UP	UP	UP
ILMN_1679262	DPYSL3 (CRMP4)	Down	Ns	Down	Down
ILMN_1720373	SLC7A5	ns	UP	UP	UP
ILMN_1751028	SERPINH1	UP	UP	ns	UP
ILMN_1768973	HIST2H2AC	UP	UP	UP	ns
ILMN_1773567	LAMA5	Down	Down	ns	Down
ILMN_1784602	CDKN1A(p21)	UP	UP	UP	ns
ILMN_1813314	HIST1H2BK	UP	UP	UP	ns
ILMN_1881909		UP	UP	ns	UP
ILMN_2144426	HIST2H2AA3	UP	UP	UP	ns

A hierarchical clustering algorithm was applied to all cells and genes using the 1 – Pearson correlation coefficient as a similarity measure. Raw data for a single array were summarized using Illumina BeadStudio v3.0 and output was a set of 43,148 values for each hybridization. We selected 13 unique genes commonly regulated in more than three p53-expressing cell lines. Univariate *t* test based on 10,000 random permutations in R packages was used to analyze differentially expressed genes. Genes with a *q* value <0.1 and a mean difference >2 were selected. ns not specific, down downregulated, up upregulated

### Transcriptional regulation of SOCS2 expression by p53

**Fig. 2:**
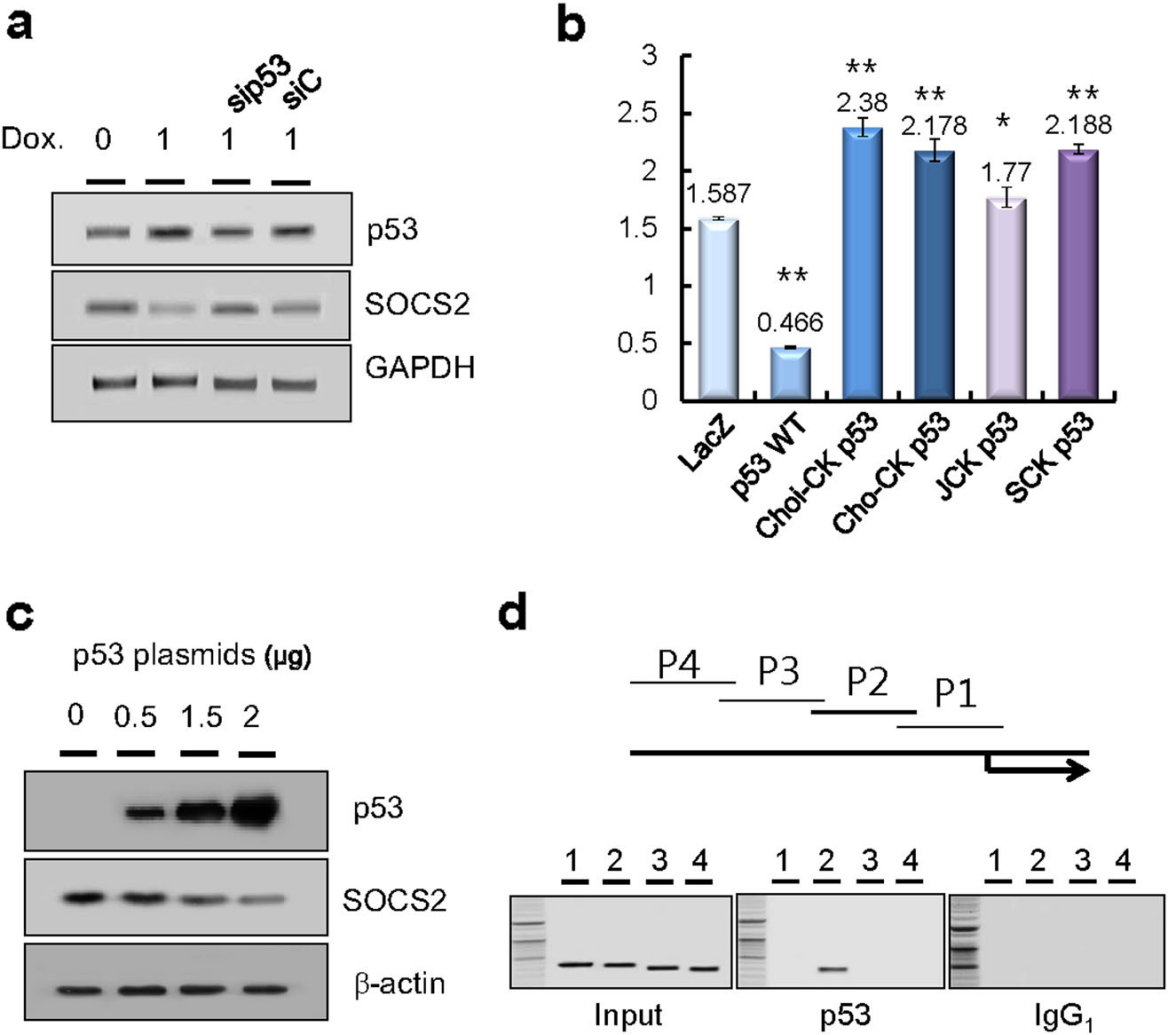
Transcriptional regulation of SOCS2 by p53. **a** Inverse regulation of SOCS2 in the HCT116 p53(+/+) cells simultaneously treated with doxorubicin and siRNA against p53 (sip53) versus control (siC). **b** SOCS2 promoter activity in wild-type or mutant p53 HEK293 cells. Values represent the mean ± SD from three independent experiments. *P < 0.05; **P < 0.01. **c** Inhibition of SOCS2 protein expression in the HCT116 p53(−/−) cells transfected with p53 expression plasmids in a dose-dependent manner. **d** Identification of p53 binding to the *SOCS2* promoter by chromatin immunoprecipitation. Crosslinked chromatin from HCT116 cells (p53^+/+^) was immunoprecipitated with anti-p53. Chromosomal fragments were amplified with *SCOCS2* primer pair P2 for 436-bp fragment in sample and no fragment in negative control (IgG_1_). NC negative control

To further investigate whether stress-mediated p53 expression downregulates SOCS2 expression, we treated HCT116 p53(+/+) cells with doxorubicin and simultaneously transfected them with siRNA of p53. We found that doxorubicin-mediated p53 expression efficiently downregulated SOCS2 mRNA expression and that p53 siRNA blocked SOCS2 suppression compared to control siRNA (Fig. 2a). Next, using a SOCS2 reporter plasmid, SOCS2 promoter activity was measured in HEK293T cells after transfection with wild-type p53 or various mutant p53 constructs (Choi-CK p53, Cho-CK p53, JCK p53, and SCK p53), which were derived from the different cholangiocarcinoma cells^20^. Wild-type p53, but not mutant p53, significantly inhibited SOCS2 promoter activity (Fig. 2b). Accordingly, SOCS2 promoter activity was higher in HCT116 (p53^−/−^) cells than in HCT116 (p53^+/−^) or HCT116 (p53^+/+^) cells (Supplementary Figure 2). Furthermore, we observed that ectopic expression of p53 by transfection with expression plasmids downregulates SOCS2 expression in a dose-dependent manner (Fig. 2c). Accordingly, promoter assays revealed that p53 effectively decreased *SOCS2* promoter activity in HCT116 cells transfected with a SOCS2-luc reporter construct with the human *SOCS2* promoter linked to a luciferase reporter gene. Chromatin immunoprecipitation assays were used to evaluate in vivo p53 binding to *SOCS2* promoter DNA in HCT116 cells (Fig. 2d). p53 bound to the promoter of *SOCS2* at the promoter site (−344 to −780 bp). We also identified potential p53-binding sequences (RRRCWWGYYY RRRCWWGYYY) within the promoter region of P2 (Supplementary Figure 3).

### Knockdown of SOCS2 suppresses tumorigenicity

**Fig. 3:**
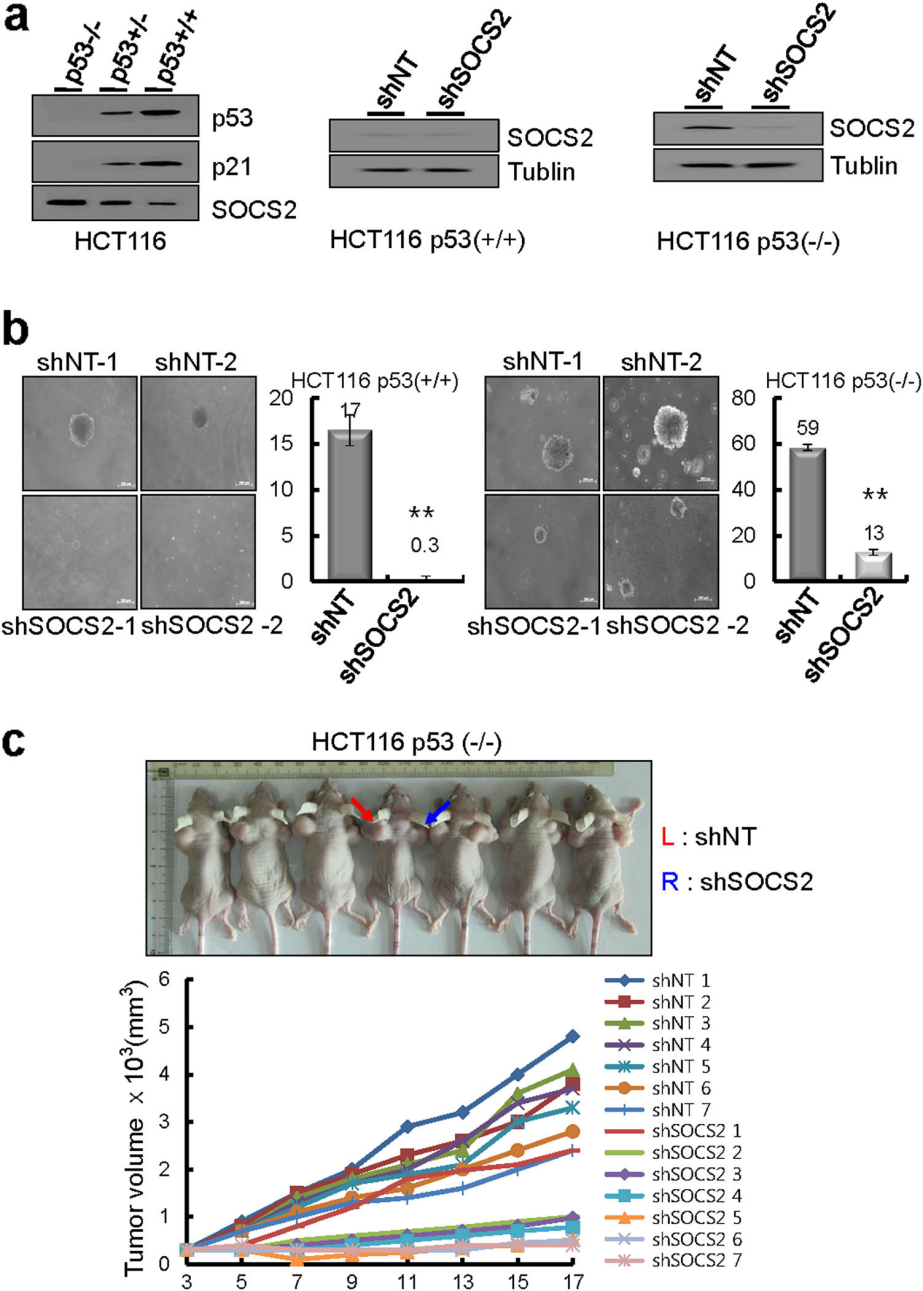
Inhibition of tumor growth by SOCS2 knockdown in p53-null cells. **a** Immunoblots from the extracts of colon cancer cells transduced with lentiviral particle encoding SOCS2 shRNA. **b** Colony formation after SOCS2 knockdown in HCT116 cells. Values represent the mean ± SD from three independent experiments. **P < 0.01. **c** Tumorigenicity after SOCS2 knockdown. Tumor cells transduced with lentiviral particle encoding SOCS2 shRNA were inoculated into right shoulders of mice and compared with inoculation of nontarget tumor cells into left shoulders (*n* = 10, **P < 0.01). Tumor growth measured at indicated times (lower panel). Values represent the mean ± SD from three independent experiments. **P < 0.01

SOCS2 knockdown cells were established in HCT116 (p53^+/+^) and HCT116 (p53^−/−^) cells through transduction with lentiviral particles encoding shRNA against SOCS2 or nontarget shRNA. SOCS2-expressing HCT116 (p53^−/−^) cells efficiently knocked down SOCS2 expression (Fig. 3a). Anchorage-independent growth of HCT116 cells was assessed using soft agar (Fig. 3b), as an indirect test of tumorigenicity. HCT116 (p53^−/−^) cells with SOCS2 knocked down formed fewer and smaller colonies (i.e., were less tumorigenic) than parental cells or control vector-transfected cells. When human HCT116 (p53^−/−^) cells with SOCS2 knocked down were subcutaneously injected into both flanks of nude mice, after 3 weeks of injections, SOCS2 knockdown cells elicited an antitumor effect, significantly suppressing tumor growth, compared to mice injected with control cells (Fig. 3c). Taken together, these data demonstrated that SOCS2 knockdown suppressed the tumorigenic capacity of HCT116 (p53^−/−^) cells in vivo, suggesting that SOCS2 may be related to tumor growth in colon cancer.

### SOCS2 expression in colon cancer tissues

**Fig. 4:**
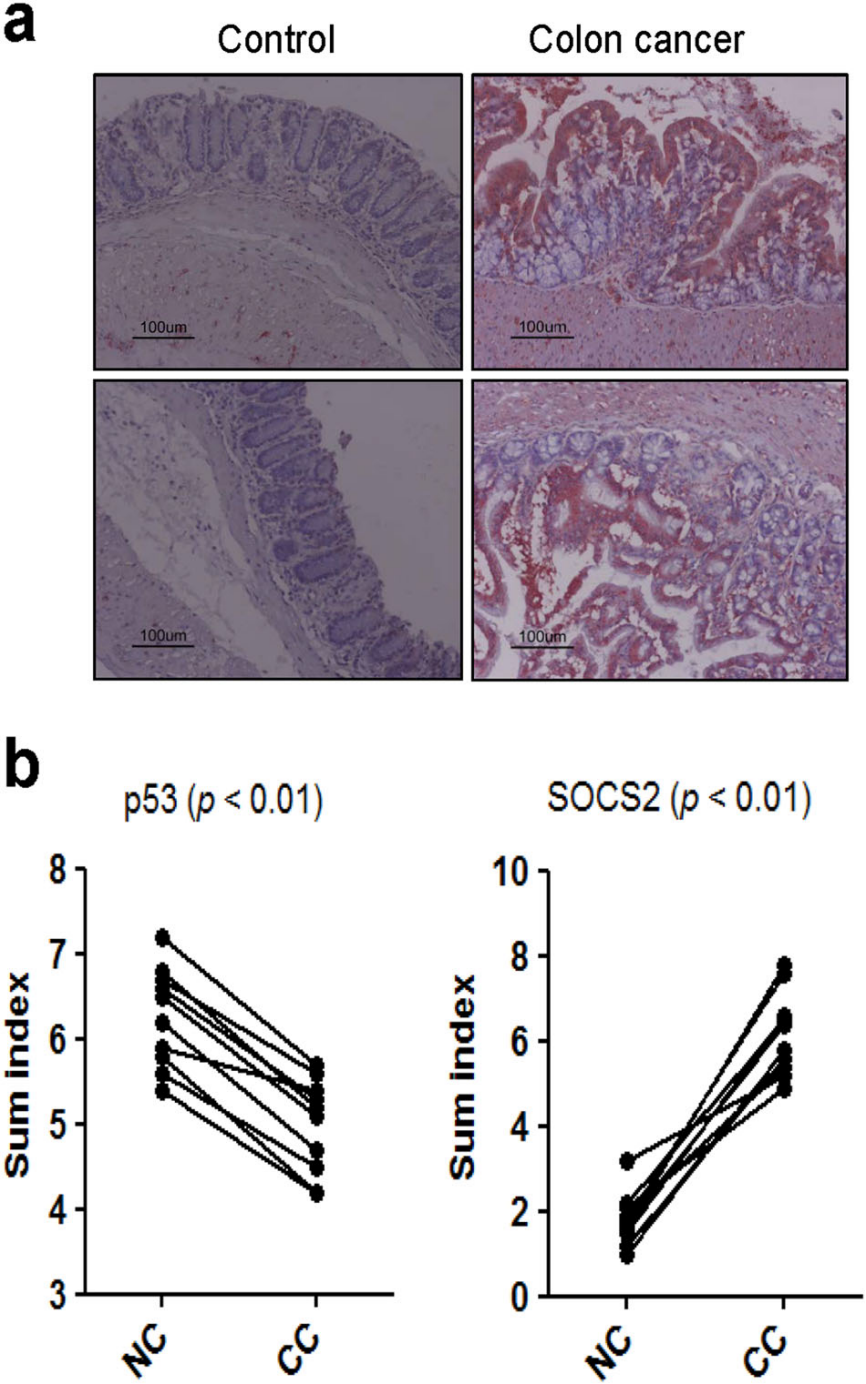
Immunohistochemistry of colon carcinoma induced by azoxymethane and dextran sulfate sodium in a mouse model. **a** Strong SOCS2 expression was detected in most colon carcinomas compared with control tissues (*n* = 10, *P* < 0.01). **b** Sum index of p53 or SOCS2 immunohistochemical staining in noncancer (NC) tissues and colon cancer (CC) tissues, respectively

**Fig. 5:**
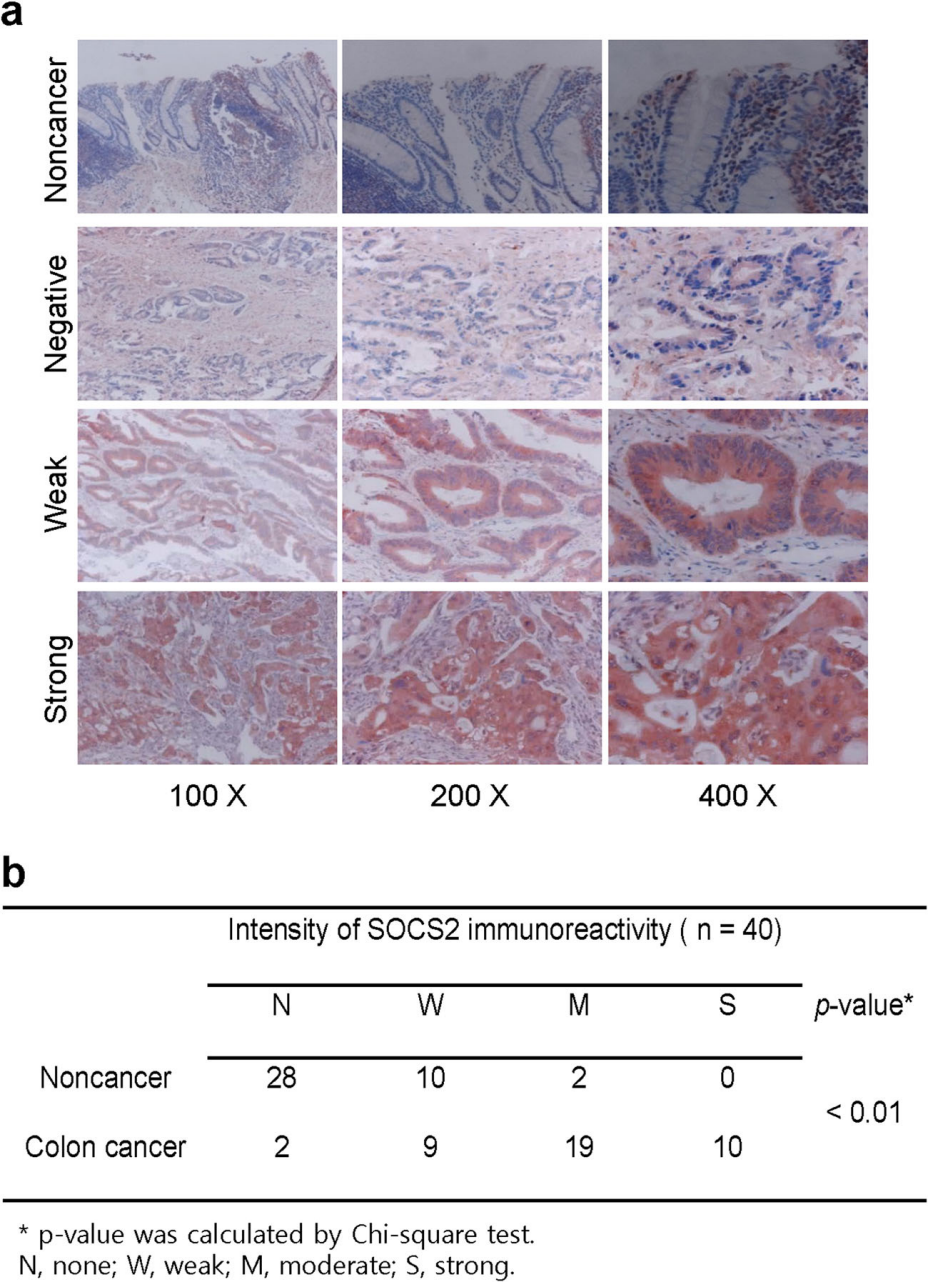
Immunohistochemistry of colon cancer tissues and corresponding nontumor tissues. **a** Various SOCS2 expression was detected in colon cancer tissues compared with the corresponding nontumor tissues (*n* = 40, *P* < 0.01). **b** Comparison of SOCS2 immunoreactivity between colon cancer (CC) tissues and noncancer (NC) tissues according to the intensity of immunoreactivity

SOCS2 expression was examined in murine colon cancer tissues derived from AOM/DSS-induced CAC samples. Strong immunoreactivity of SOCS2 was mostly found in the cytoplasm of CAC tissues compared to control colon tissues (Fig. 4a). Immunoreactivity for p53 was significantly reduced in colon cancer tissues compared to noncancer tissues, whereas SOCS2 immunoreactivity was enhanced in colon cancer tissues (*n* = 10, *P* < 0.01, Fig. 4b). In human colon cancer tissues, SOCS2 immunoreactivity was heterogeneously detected. However, most colon cancer samples showed upregulated SOCS2 expression (*n* = 40, *P* < 0.01, Fig. 5a). SOCS2 immunoreactivity was significantly stronger in colon cancer tissues than in noncancer tissues (*P* < 0.01), as shown in Fig. 5b.

## Discussion

SOCS family members were initially identified as negative regulators of cytokine signaling through JAK and STAT signaling^21^. Activated STATs translocate into the nucleus and mediate gene transcription and regulate cell survival and transformation^22^. Cytokine receptor-mediated JAK/STAT activation leads to SOCS induction. This suppresses cytokine signaling by inhibiting JAK activation, competing with STATs for binding sites on cytokine receptors and by targeting signaling proteins for proteosomal degradation ^21^.

Downregulation of SOCS2 enhances proliferative and antiapoptotic actions of IGF-I in the small intestine and colon and the ability of IGF-I to activate STAT3 and negatively regulate aberrant intestinal growth in a model of GH and IGF-I excess^23^. High SOCS2 expression is inversely correlated with breast cancer tumor grade and positively correlated with a good prognosis^24^. Breast carcinoma studies have demonstrated that SOCS2 protein expression is also positively correlated with low-grade tumors^25^. These studies in primary human tumors suggest a potential role for SOCS2 in suppressing tumor growth. SOCS2 is also recognized as a tumor suppressor because reduced expression upon hypermethylation is associated with activation of STAT3 in ovarian and breast cancers, indicating increased cytokine responsiveness in tumors^26^. In contrast, SOCS2 is a pro-oncogene in advanced stages of chronic myeloid leukemia and in precursors of anal cancer, where it is significantly upregulated^12,13^. Differences between SOCS2 mRNA and protein levels are observed in cells as a consequence of active degradation of SOCS2 protein^27^. In tissue samples, SOCS2 is increased in malignant areas and SOCS2 expression positively correlates with increased Gleason scores in prostate cancer^14^. *SOCS2* is an androgen-regulated gene and elevated levels in prostate cancer are consistently observed in independent patient cohorts.

In concordance with cell expression profiles, our experiments indicated the potential growth-promoting activity of SOCS2 in colon cancer. First, SOCS2 was a negatively regulated target gene of p53. Second, SOCS2 overexpression led to significantly increased tumor growth in vivo. Third, *SOCS2* knockdown substantially decreased cell growth and tumorigenicity of colon cancer cell lines.

Our hypothesis regarding SOCS2 as a growth promoter rather than an inhibitor is consistent with data from a prostate cancer study^14^. Both SOCS2 knockout and transgenic mice display gigantism^17,28^. This suggests that SOCS2 has dual functions in growth regulation, depending on its concentration. At low levels, SOCS2 inhibits cascades such as GH, prolactin, and IL signaling. At high levels, SOCS2 restores or potentiates responsiveness to these growth factors^29–32^. Our data showed that p53 induction suppressed ectopic expression of SOCS2, suggesting that p53 expression might be associated with SOCS2 degradation in PC3 and HLK3 cells. Suppression of SOCS2 expression might be associated with inhibition of tumor cell proliferation. Therefore, we hypothesized that SOCS2 expression is involved in tumor cell growth.

In this study, wild-type p53 efficiently suppressed SOCS2 expression, but mutant p53 did not. Chromatin immunoprecipitation assays revealed that wild-type p53 binds directly to the promoter area of the *SOCS2* gene. P53 inversely regulated SOCS2 expression in HCT116 colon cancer cell lines according to functional p53 (p53^−/−^, p53^+/−^, or p53^−/−^). In other colon cancer cell lines, SOCS2 expression was reciprocally reduced by functional p53 expression. Thus, SOCS2 expression could be subject to transcriptional and posttranscriptional regulation by p53. In in vivo animal models, SOCS2 knockdown decreased tumorigenesis. Furthermore, strong immunoreactivity for SOCS2 antigen was observed in colon carcinomas induced by azoxymethane and dextran sulfate sodium. However, varying expression of SOCS2 was seen in human colon cancer tissues. This discrepancy might be because of differences in the oncogenic process between chemically induced and genetic mutation-mediated backgrounds.

In summary, SOCS2 appears to be a target molecule that is negatively regulated by p53. SOCS2 expression causes tumor growth and progression, which could determine colon cancer prognosis. Therefore, the p53/SOCS2 signaling pathway may be a useful target for colon cancer chemotherapy.

## Electronic supplementary material

Supplemental Figures
