# Supplementary material for: Alterations in the p53-SOCS2 axis contribute to tumor growth in colon cancer
Source: Exp Mol Med. 2018 Apr 6;50(4):3. doi: 10.1038/s12276-017-0001-1 (PMC5940812; doi:10.1038/s12276-017-0001-1)
Supplement: Supplementary file 1 — Supplemental Figures [file 12276_2017_1_MOESM1_ESM.pdf]

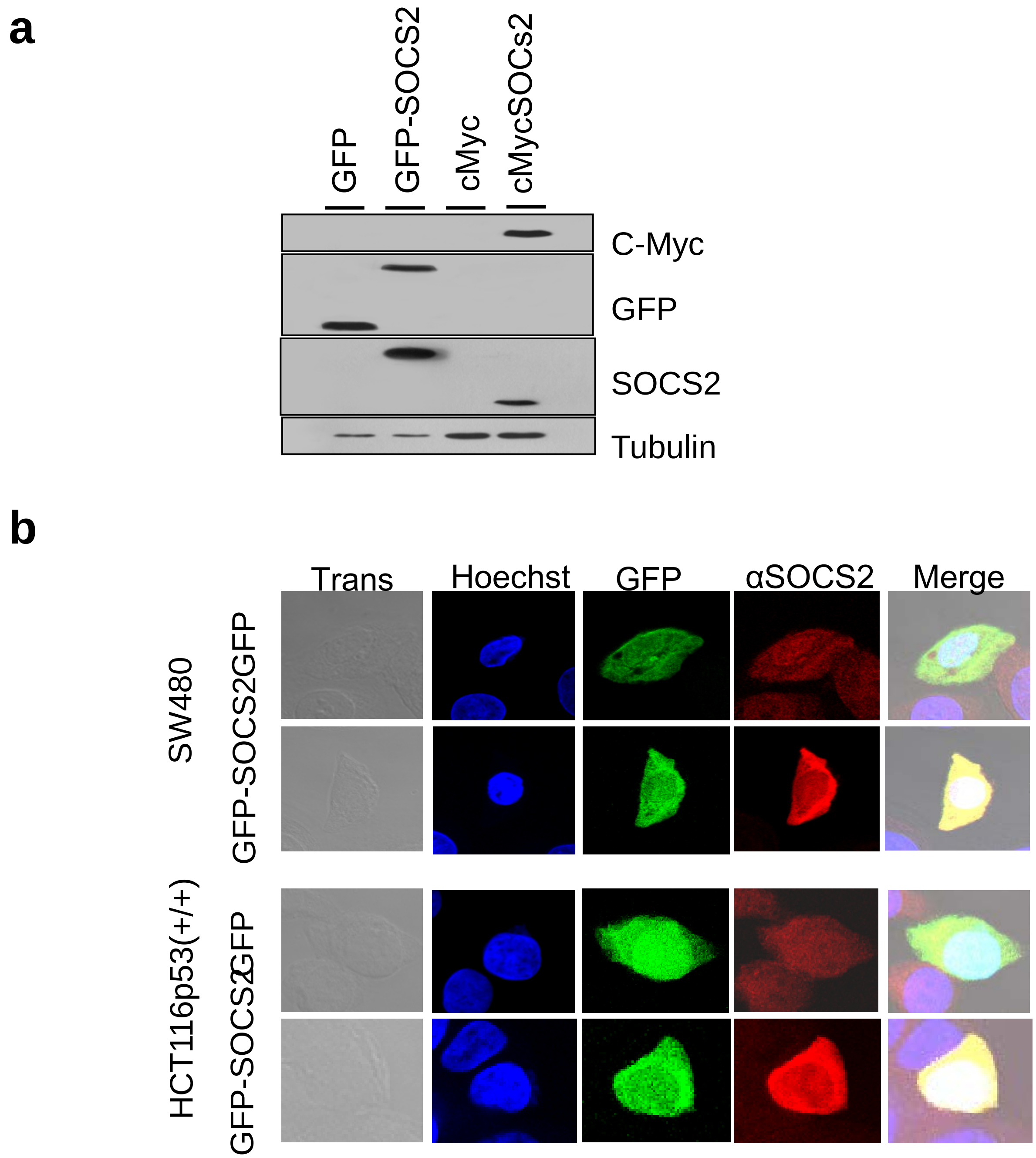

Figure S1. Cellular localization of SCOS2. (a) Specificity of SOCS2 antibody in H293T cells transfected with GFP- or cMyc-tagged SOCS2 expression plasmids. Immunoblots of cell lysates. (b) SOCS2 in cytoplasm and nucleus of colon cancer cells. Immunofluorescence of colocalization of GFP-tagged SOCS2 and anti-SOCS2 in colon cancer cells.

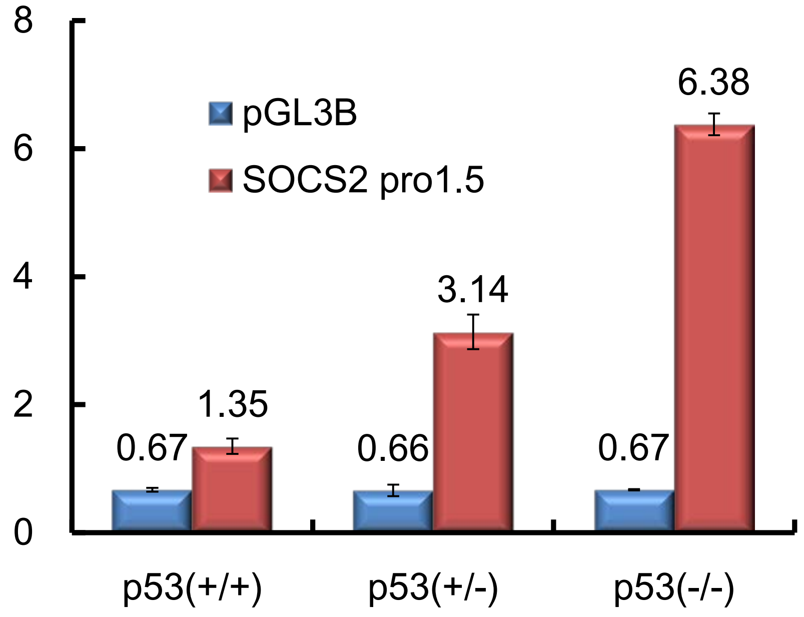

Figure S2. SOCS2 promoter activity of HEK293 cells according to p53 status (p53 -/-, +/-, or +/+). Values represent the mean ± SD from three independent experiments. *P < 0.05; **P < 0.01.

```
                         gtaatcccagcttctcgggaggctgaggca
ggagaattgcttgaacctgggaggcggaggttgcagtgagctgagatcac
gccctgcactccagcctgggcgacagagggagacttggtcatgctccctc
cccgccctccgtcagttttaggaataaatacctttttatttaagctaaag
tgtgggtacacccttcctctaggattctccatcaaggaataagaagccat
attaggacaatttagagggcagttaaccctagtagacatagtggttctta
aaaggcttggggcctcagactgtacacaggcttcacatggaatctgattt
gttcctttatcccagtccctcacccagaacccgaatctagcccttcatgt
tataaaaagggccagaggtccaaagagggtaagtgccttgtgcaaaatta
ttcaac
```

Figure S3. Potential binding sequences to the consensus p53 binding sequence (RRR**C**WW**G**YYYRRR**C**WW**G**YYY; R, purine; W, A or T; Y, pyrimidine) in the P2 promoter region (-344 to -780 bp) of SOCS2.
